# Traditional ecological knowledge and flood risk management: A preliminary case study of the Rwenzori

**DOI:** 10.4102/jamba.v10i1.536

**Published:** 2018-05-31

**Authors:** Bosco Bwambale, Moses Muhumuza, Martine Nyeko

**Affiliations:** 1 Faculty of Agriculture and Environment, Gulu University, Uganda; 2 School of Agriculture and Environmental Sciences, Mountains of the Moon University, Uganda; 3 Centre for Action and Applied Research for Development, Fort-portal, Uganda

## Abstract

The shift from flood protection to flood risk management, together with recent arguments on incorporating culture in managing risk, underscores the application of traditional ecological knowledge (TEK) in managing disasters from flood hazards. Yet, documentation and incorporation of TEK into practice remains a challenge. This article contributes to addressing this challenge by exploring the existence of TEK to flooding in the Rwenzori Mountains, Uganda. Using semi-structured interviews, data were collected from residents of the Nyamwamba watershed where intense flash floods caused deadly impacts in May 2013. Collected data were analysed using content, thematic and interpretive analysis techniques. Results indicate that TEK is exhibited through various traditional ecological approaches (TEAs). Although endangered, TEAs (conducted through collective action for a communally accepted end) are framed in three main activities: (1) assessment and prediction of rainfall and flood by the traditional hydro-meteorologist (*diviner*) and the traditional rain forecaster (*rainmaker*); (2) the mountain cleansing ritual (which act as flood risk awareness platform); and (3) immunising riverine communities through planting certain indigenous plants, which improve hydrological systems through their high conservation value for native ecological diversity. As most TEAs are conducted through collective action, they represent a platform to understand local capacities and enhance adoption of measures, and/or a source of knowledge for new measures to address flood risk. Therefore, full-scale investigations of these TEAs, determining how relevant TEAs are fine-tuned, and (scientific) measures enculturated based on fine-tuned TEAs could result in effective flood risk management in various flood hotspots where TEAs influence action.

## Introduction

With the increasing emphasis on building resilience to natural hazards (UNISDR 2015), together with the recent focus on the role of culture in disaster risk management (IFRC 2014), approaches linked with traditional ecological knowledge (TEK) will become more relevant. Traditional ecological knowledge is that sort of ‘indigenous knowledge’^1^ that originates from interactions between indigenous people and their ecosystems (Iloka 2016; Tran et al. 2008; Vogel et al. 2007). As human interaction with ecosystems is hardly restricted to specific ecosystems, TEK also refers to ‘traditional environmental knowledge’ signifying that knowledge is used by humans to connect with the complex web of their local environment (Houde 2007). Especially for hydro-meteorological hazards (especially floods), given their occurrence often during rainy seasons (Trenberth 2011), traditional riverine communities have a regular interaction with flood events and hydrological systems.

Through interactions with their hydrological systems, indigenous people accumulate intimate perceptions and experiences through observation, sharing, monitoring and evaluation of how flood hazards progress into disasters (Iloka 2016; McEwen & Jones 2012). In this process, they get acquainted with concrete knowledge and evolve practices or approaches of how and where to place certain enterprises or plant species that have proven capabilities to resist flood events while enhancing the livelihoods and benefits associated with flooding (Iloka 2016). Therefore, their views on what is likely to work become essential to consider in assessing and reducing the impacts and risk due to floods events, and, importantly to enhance flood risk management (FRM).

Flood risk management is the contemporary paradigm in hazard and disaster risk management. It emerged after realisation of (1) the historic and inexorable utilisation of the precious riverine ecosystem resources for socio-economic development (Hyndman & Hyndman 2014; Oxley 2011; Smith & Petley 2009); (2) the cultural attachments (to the water resources) which motivate occupation around flood hazard zones (Cannon, 2015; IFRC 2014); and (3) the residual risk which could not completely be addressed through the protectionist paradigm which relied mostly on structural approaches to tame flood processes (Heidari 2009; Khan 2012; Singkran 2017). Contemporary understanding rather finds that natural hazards like floods will turn into disasters depending on the resilience of the exposed communities (Smith & Petley 2009; Wisner et al., 2004). For instance, developed countries (such as Japan) are exposed to various environmental hazards, but experience limited damages and losses because of their (sophisticated) coping mechanisms (Smith & Petley 2009). This implies that effective reduction of risk not only means taming the hazard, but as well enhancing capacities of the communities-at-risk to become resilient to disaster (Cutter et al. 2008; Oxley 2011; Smith & Petley 2009).

The concept of resilience to flood disaster(s) underscores the application of TEK. Linked with TEK are the various traditional ecological approaches (TEAs), which are creative practices employed by communities-at-risk to deal with flood disaster. The motive to devise TEAs to flooding underscores, firstly, the awareness or perception of flood risk by the communities-at-risk and, secondly, the belief in their abilities to manage and/or reduce the risk and so is the possibility to implement measures (Iloka 2016; Jha & Jha 2011; Smith & Petley 2009; Tran et al. 2008; Vogel et al. 2007). This means TEK is both a knowledge source (for approaches to address floods) and a platform upon which scientific measures can be introduced into the communities-at-risk (which are key players in implementing measures). Yet, in practice, TEK and TEAs are still ignored. The two common reasons for ignoring this knowledge include the following: (1) such kind of knowledge is hardly documented, and (2) ways of how it can be fine-tuned and used (together with scientific measures) are rarely well-articulated (Iloka 2016; Jha & Jha 2011; McEwen & Jones 2012).

**FIGURE 1:**
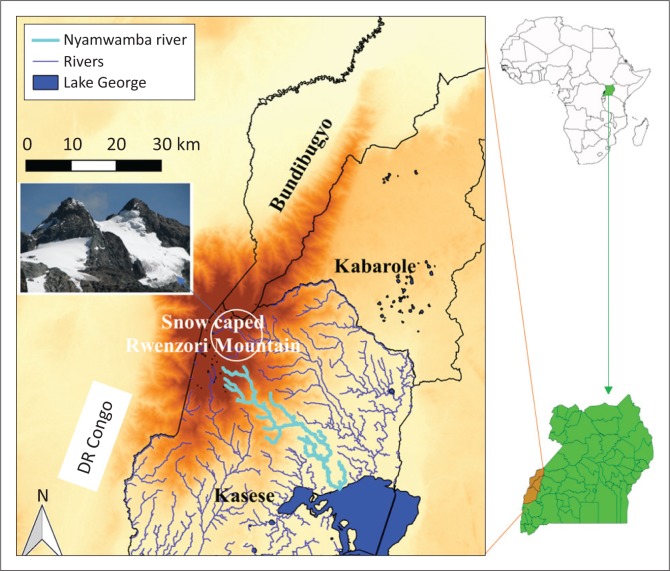
Location of Nyamwamba River in south-western Uganda (Africa).

**FIGURE 2:**
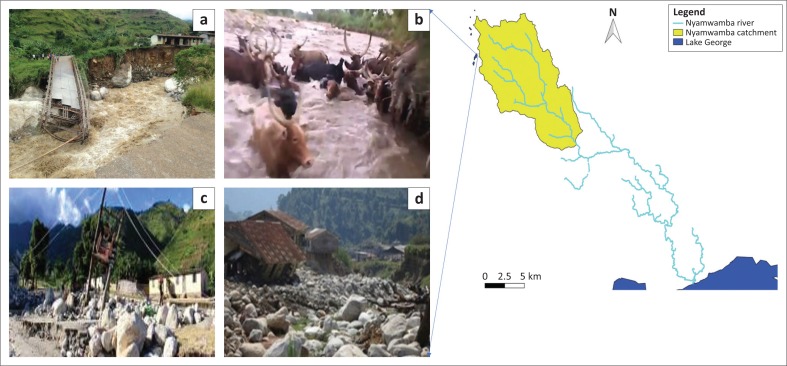
Sample damages caused by floods in the Nyamwamba watershed; pictures are from the Nyamwamba catchment on the Nyamwamba watershed. (a) Destruction of bridges, (b) cows trapped in flood, (c) destruction of live lines and (d) destruction of residential buildings.

This study contributes to the documentation of and the ongoing scholarly debate on incorporating TEK in FRM. More specifically, the study was conducted (1) to explore the existence of TEK and (2) to highlight TEAs that might be relevant to flood risk science and effective FRM in the Nyamwamba watershed (Rwenzori Mountain Region, Uganda). In this watershed (Figure 1), flooding has resulted in several recent disasters with the highest losses per event in the country (Jacobs et al. 2016, 2017; Kitutu 2013; UNDP 2013; Figure 2). Yet, in this watershed, recommended riverbank management interventions (Kitutu 2013) to reduce flood impacts have continuously been rejected by the communities-at-risk (Jacobs et al. 2016). Considering that flooding is not a new phenomenon in this community (UNDP 2013), it is relevant to understand the flood experience of this community and use their experience to guide effective FRM.

## Research methodology

### Study area description

This study was carried out along the Nyamwamba watershed, the hotspot in the Rwenzori Mountain Region, Uganda. The watershed covers 326 km^2^ and lies within the Lake Victoria Basin (Eggermont, Van Damme & Russell 2009; Kabenge et al. 2017). An estimate of 126 km is stretched by the Nyamwamba River with its tributaries, pouring into the Lake George (Figure 1). On the Rwenzori Mountains, where the Nyamwamba River originates, glaciers also exist (Figure 1), and although the Nyamwamba River does not drain the glacial region (Jacobs et al. 2016), glacial processes constitute part of the climatological processes of the Rwenzori Mountain Region (Taylor et al. 2006).

Two annual rainfall seasons (August to December and March to May) trigger landslides and floods (Eggermont et al. 2009; Jacobs et al. 2015; UNDP 2013), especially for floods (Kitutu 2013; UNDP 2013). Given its mountains nature, flooding in the Rwenzori takes the form of flash floods and so is the reason it constitutes deadly and high impacts to the inhabitants (Jacobs et al. 2016). (see Figure 2)

Whereas the severest (01 May 2013) flood event in this watershed was reported as unprecedented (Jacobs et al. 2016), a historic rise in intensity and frequency of flood events along the Nyamwamba and other Rwenzori watersheds is also reported (UNDP 2013). Alongside these historic events, specific categories (usually men) among the Bakonzo ethnic group (who form the main native group) are reported to hold specific cultural positions in the management of hazards in various hills in the Rwenzori Mountains (De Hontheim 2015; Musoke 2015). As cultural practices are an indicator of what the communities-at-risk are likely to adopt (Cannon 2015; Jha & Jha 2011), and considering that modern flood measures are frequently rejected in the Rwenzori (Jacobs et al. 2016), it is relevant to understand the traditional flood experience of this community and use it as a knowledge or a platform for effective FRM.

### Materials and methods

To achieve the objective of this study, which was to investigate the existence of TEK to flooding in the Rwenzori, a qualitative research approach was employed. Although this approach allows for small purposive samples (Clifford et al. 2016; DiCicco-Bloom & Crabtree 2006), this was insignificant compared to its contributions to this study. The study required an in-depth exploration and understanding of the existence of TEK and the TEAs linked to it, and if in anyway it would be significant to flood risk science and effective FRM. Specifically, (individual) semi-structured interviews were conducted on a purposive sample along the Nyamwamba River flood-hotspot zone in the Rwenzori, Uganda (Figure 1). Semi-structured interviews are relevant in studies where in-depth exploration is requested from participants, and especially for those who (by one reason or another) may not be comfortable enough to talk in the presence of others (Bernard 2006; DiCicco-Bloom & Crabtree 2006). Accordingly, as Christian religion tends to discourage practices related to TEK (De Hontheim 2015), semi-structured interviews were used under the premise that some participants might fear to talk about them in the presence of others. Besides, the semi-structured character of these interviews also allows an open non-suggestive coverage of the topic of inquiry.

Selection of the purposive sample was guided by the Ministry in Charge of ‘Ethics, Integrity, Disasters and Regional Recovery’ in the local cultural institution, ‘Obusinga Bwa Rwenzururu’ (De Hontheim 2015). This institution, being promoters of traditional practices in the region (De Hontheim 2015), had knowledge and records of people knowledgeable about TEAs to flooding in the watershed.

As this study required the most knowledgeable participants, some inclusion criteria were further added. Participants were included based on a twofold criterion: (1) being a resident of the community-at-risk of flooding (Nyamwamba watershed in particular) from the time of birth (till the time the study was conducted) and (2) minimum age of 50 years. In both, it was to ensure that the participants are those with adequate historic account of flood dynamics and traditional practices in the area. Based on these criteria, 10 participants were selected for the interviews.

Between 08 March 2017 and 30 March 2017, an interview guide was administered to the 10 participants. The guide included questions on four main themes: (1) the historic practices employed by the communities-at-risk to address flood disasters, (2) the historic practices which are still practised, (3) the historic practices which are no longer practised and (4) understanding the reasons for those that are no longer practised. Field noting and audio recording were used to capture the data during interviews. After that, the collected data were transcribed, coded and sorted for analysis.

The content, thematic and interpretive analyses were used to analyse the data. Working through the transcribed data, codes were assigned to the various homogenous categories in the text. These guided the creation of themes for the interpretive analysis. The analysis of the results was guided by the question: ‘Which TEAs, in the Rwenzori, could be further studied, fine-tuned and incorporated into the modern FRM?’

## Results

Consistent with the themes derived from the analysis, the TEK to flooding in the Rwenzori is arranged under four main categories of TEAs: firstly, the historic TEAs to flood events (including the rituals and the planting of cultural flood-immunising indigenous plants); secondly, the TEAs in practice; thirdly, TEAs no longer practiced; and fourthly, understanding reasons for (not) practising some TEAs (Tables 1 and 2).

**TABLE 1:** Status of traditional ecological responses.

Practice	Details
*Eri’hera*	Done, to a limited extent, by some spiritual media [*a’bathahwa*].
*Eri’honga*	No longer practised, but the cultural institution (Obusinga Bwa Rwenzururu) plans to revitalise it.
Planting indigenous plants	Still practised by some individuals along the Nyamwamba River.

**TABLE 2:** Verbatim description of factors that could have threatened traditional ecological approaches to flooding in the Rwenzori.

Factor	Description (verbatim)
(Christian) religion	‘Religion has monopolized the whole system; people feel shy to talk about these practices as they do not wish to be identified as against the God of Christianity [who is taken to be superior to the other gods that traditional religions used to revere]’. (Interviewee C, personal interview, 13 March 2017)
Gazetting of the Rwenzori Mountains National Park	‘Most of the practices were done deep in[side] the forest [Rwenzori Mountain]; it is now gazetted and under the park [Rwenzori Mountains National Park]; anybody found there is thought to have broken the law, and some people have been shot dead because of this’. (Interviewee J, personal interview, 28 March 2017). ‘Even when they permit some cultural practices inside the park, they want to know what the practice is about, yet some rituals do not allow for informing anybody’. (Interviewee C, personal interview, 13 March 2017)
Death of some practitioners	‘Some people who used to carry out these activities, such as *a’bathahwa*, have died at a rate higher than the pace at which they could train some other people to succeed them’. (Interviewee C, personal interview, 13 March 2017)
Destabilisation of the cultural institutions	‘The cultural institutions were abolished [destabilised] during the regime of Obote; [therefore] the traditional practitioners laid down their tools’. (Interviewee F, personal interview, 17 March 2017)

### Historic traditional ecological approaches to flood risk management in the Rwenzori

#### The individual cleansing ritual, *Eri’hera*

According to the participants, *Eri’hera* is a cleansing ritual conducted by a spiritual medium or seer or diviner [*o’muthahwa*] or a designated person to claim justice or return tranquillity. Regarding extreme environmental events, *Eri’hera* practice consists of two main activities: (1) the act of traditional rain-making and (2) the acts by *a’bathahwa* (the spiritual mediums, also synonymous to native doctors and/or diviners).

The activity of traditional rain-making, according to participants, is one where the *‘o’muhangyi* [rainmaker] makes rituals to provide sunshine or rain with power bestowed by an ancestral spirit’ (Interviewee A, personal interview, 09 March 2017). The rainmaker would be consulted to provide sunshine in case of too much rainfall that would be thought capable of causing flood or to provide rainfall in case of too much sunshine possible to cause drought.

The acts by *a’bathahwa* [spiritual mediums] involved intercession, where the spiritual medium (*o’muthahwa*) along the Nyamwamba River would study river dynamics to detect an upcoming flood events and accordingly immunise the river against it (*eri’tsirika*). Quite often, it was mentioned ‘he [*o’muthahwa*] prepared certain concoctions, and threw them into the river and planted specific plant species at specific points of the river to prevent similar calamity in the future’ (Interviewees A, B, personal interview, 11 March 2017).

#### The communal cleansing ritual, *Eri’honga*

Participants defined the term *Eri’honga* as a communal cleansing ritual organised to prevent or mitigate disasters (such as drought, famine, floods and landslides): *Eri’honga* is similar to *Eri’hera*. However, the former is communal, in contrast to the latter which was performed by a designated individual (although most frequently demanded by and conducted on behalf of the community for a communal good).

In the *Eri’honga* ritual, the overall goal is to return tranquillity to the disturbed community or to prevent disturbance in case a disaster was predicted. The frequently mentioned activity in this practice was *eri’birya a’malhambo*. In the literal sense, according to the participants, ‘*Eri’birya a’malhambo*’ means sweeping (*eri’birya*) the ridges and/or villages (*a’malhambo*); the connation of this practice is cleansing of ridges to chase away bad spirits, *e’mirimu mibi,* which are responsible for catastrophes. Catastrophe can, according to participants, also occur if *e’mirimu ye’kithwa* [the gods of the mountain] are annoyed or disturbed by human activities. This would justify the regular ritual, *eri’hongera e’mirimu* [appeasing the spirits] through mountain cleansing.

According to the participants, during mountain cleansing, the ridge leader (a chieftain, [*o’mukulhu wa’bulhambo*]) leads the community people from the hills to the lowlands. While moving, certain songs are chanted and meats of certain sacrificed animals are thrown on the pieces of land along the path. In the entire exercise, words referring to certain spirits would be mentioned by [the] *o’mukulhu wa’bulhambo* or any designated diviner to appease the concerned spirit. Regarding flooding, it was the spirit called *dyoka* (the mystic octopus regarded as god of the rivers) that was appeased. In a way of preventing or mitigating floods, most of the participants concurred to the position that:

‘The practice [used to] start in the hills up, with slaughtering of certain animals and preparing some concoctions.’ (Interviewee D, personal interview, 13 March 2017)

‘…blood from slaughtered animals is also sprinkled along the way; some of the meats from the slaughtered animals are eaten by the participants and others are thrown along the way in the procession, and the practice ends down in the low land, chanting songs specific to the calamity.’ (Interviewee G, personal interview., 23 March 2017)

‘… to chase away the bad spirits that cause it’. (Interviewee C, personal interview., 13 March 2017)

#### The planting of culturally flood-immunising indigenous plants

According to the participants, in addition to the rituals, some indigenous plants are identified and planted along the river and watershed as flood immunisers. The frequently mentioned indigenous plants were: *esya’mbathi* [the bamboo, *arundinaria alpina*], *o’mulingatha* [*erythrina excelsa*], *o’muramura* [*dracaena fragrans*], *o’muthubya* [*cordia africana*], *a’maranga* [*Neoboutonia macrocalyx*] and *e’binyangwa* [the yams, *Dioscorea Villosa*]. These were among the respected plants and were planted along the rivers to prevent and buffer communities (along the rivers) against adversities of flooding. ‘Whoever was found cutting such trees was taken to e’Kyikali [the palace] for caution’ (Interviewee H, personal interview, 23 March 2017) ‘or to answer why they did it’ (Interviewee C, personal interview, 13 March 2017).

### Traditional ecological approaches to flood risk management in practice

Participants frequently mentioned that all TEAs are threatened and are at a standstill (Table 1). They, however, frequently mentioned that there are some cases where some of these practices are still recoursed to albeit, sometimes, with people that are not qualified. Frequently mentioned was the view that: ‘those doing them these days are looking for survival; they are not traditionally recognised [meaning not qualified] for those practices’. (Interviewee D, personal interview, 13 March 2017). To identify which measures are still recoursed to or not, participants were asked about the state of each measure (Table 1).

### Practices no longer in existence and responsible factors

Data from the participants agreed to the view, in general, that most of the TEAs are not being practised (Table 1). Table 2 indicates that religion, gazetting of the Rwenzori Mountains National Park, the rapid death of the traditional practitioners and the abolishment of cultural institutions are the main factors that contributed to the gradual disappearance of the TEAs to FRM in the Rwenzori.

Furthermore, regarding subjective opinions on whether revitalisation of some measures would contribute to FRM, participants frequently mentioned:

‘[…] they [TEAs] can still help […] we have changed to a religion alien to ours; we are now practicing their [missionaries’] culture (Interviewee J, personal interview, 28 March 2017)

‘… we have moved away from our culture to their culture […] our culture can still work for us’. (Interviewee C, personal interview, 13 March 2017)

## Discussions

### Overview

This study focused on exhibiting the existence of TEK to reducing the impact and risk of flooding, and highlighting the TEAs linked to it that might be relevant for effective FRM in the Rwenzori Mountain Region. The analysis and interpretation of responses from the participants reveal the two cleansing rituals (*Eri’hera* and *Eri’honga*) and the planting of cultural immunising plants along the riverbanks as the main TEAs to flooding. These practices are, however, threatened. The main factors cited as threats against TEK (and TEAs linked to it) include: (1) the Christian religious campaigns against it, (2) the conservationism expressed in the gazetting of the Rwenzori Mountains National Park, (3) the (rapid) death of the traditional practitioners and (4) the destabilisation of the cultural institutions (but only in some period in the period). Considering that the cultural institution plans to revitalise the TEAs (Table 2), the main threats remain religion and conservationism.

### Traditional flood risk management and modern flood risk science

While most of the TEAs to flooding are at standstill (and threatened), (1) interest and/or tribute is still paid to them, and (2) they can be reconstructed by some members in the community. This means that in case they are found to effectively reduce the risk of flooding, they would easily be revitalised and incorporated into both the modern flood risk science and the modern measures to address (adverse) impacts of flood events. Of course, the question remains on how these TEAs can be proven to be effective when they appear as beliefs. As belief systems indicate what communities are likely to adopt (Cannon 2015; Jha & Jha 2011), they can as well be used as a guide on what to consider when introducing other (scientific) measures in traditional communities (IFRC 2014). Moreover, in some African traditional communities either traditional beliefs are quickly recoursed to when modern measures seem inadequate (Maathai 2010) or in some communities traditional beliefs and approaches have consistently existed alongside the modern approaches (Muhumuza 2015).

The interest in and the reconstruction of the TEK observed reveal the persistence of and the importance TEAs could still have within the Rwenzori: despite the campaigns (and other cited factors) against them, TEAs are still of interest to the people that hold belief in them. Such is the role that culture can play in disaster risk management (Cannon 2015) and the fact that some once culturally alienated Africans are noted of regressing back to their traditional ways of life (Maathai 2010).

In general, the described TEAs indicate a clear mode of traditional FRM which can be relevant for scientific FRM. In the scientific perspective, FRM constitutes of three main strands: (1) risk assessment, which involves assessing the hazard and the capacity of those exposed; (2) risk reduction, which involves identifying and implementing certain measures; and (3) response and recovery, which involves the rescue and relief during and after disasters, respectively – overall, FRM aims at reducing the risks and/or threats and maximising any benefits related to hazards (Smith & Petley 2009). Regarding these findings, (traditional) FRM is related in two main practices: firstly, the actions by the traditional ecological scientists (i.e. the seer and/or diviners and rain-makers), who assess weather and use or propose certain practices or planting of certain plants to avert any catastrophe; and secondly, measures through the exercise of the planting of certain cultural immunising indigenous plants, and the mountain cleansing. The rainmaker and diviner could be related to the meteorologists and hydrologist who forecast and/or predict rainfall and flood events based on the weather patterns. The planting of indigenous plants is already relevant to flood risk science where native tree species are reported to fortify riverine communities against floods (Bradshaw et al. 2007). The cleansing ritual perhaps acted as a means of raising flood awareness, enhancing preparedness and community service. While these traditional practices would need further investigation, two main inferences are clear about them: firstly, the traditional and communal self-reliance where communities believed in their abilities to assess and manage extreme environmental events; and secondly, the use of spatial (context-specific environmental) resources as measures to address threats posed by those events. The following subsections delve on each of these and their link with flood risk science: firstly, the traditional resilience; secondly, the use of environmental resources; thirdly, the cleansing rituals and indigenous plants; and finally, a discourse on religion and conservationism with regards to FRM.

### Traditional resilience to flooding and contemporary flood risk science

A socio-cultural attachment to water resources (and socio-cultural practices to manage those resources) is eminent. The motive seems not only managing the flood hazard but also ensuring that the water resources are not exploited by extremes in environmental conditions. This motive is exemplified in the hierarchical framework linked to the cultural institutional administration of the water resources. This cultural administration is recognised as an arbitrating authority for any deviant behaviour to the management of hydrological resources (e.g. for cutting or not planting cultural flooding immunising plants). This manifests the value that the community attached to the hydrological resources. Such a socio-hydrological orientation to addressing flooding is highlighted by various scholars (Fuchs et al. 2017; Karamouz, Szidarovszky & Zahraie 2003) as a necessary factor to consider in the management of water resources of which managing flood risk is a part. According to these scholars, a historic socio-hydrological approach to managing water resources indicates the historic community adaptation to the hydrological hazards, and more specifically, the necessary adjustments and preparation made by the members of a community on how to deal with disasters associated with hydrological hazards. Accordingly, it underscores the community creative reasoning about flooding, their perception and interpretation of the impact and risk floods (can) pose, and most importantly, what the local communities are likely to do with regard to reducing the impact, recovering, planning and preparedness for flood hazards (Cannon 2015; Fuchs et al. 2017).

The described traditional resilience to hazards through the TEAs seems a score on the part of this community. However, it also underscores the readiness of the community to appreciate measures that hardly compromise this status quo. More specifically, resistance from such a community might arise if such traditional abilities are confronted (Kottak 2009) by modern scientific approaches of managing flood risk. This, however, means that paying attention to these traditional ecological ways of life could provide an incentive to effective implementation of measures to address floods at the local levels (IFRC 2014; Maathai 2010). However, as traditional ways of life are diverse, the focal point of the traditional community would be the suited guide. Regarding this community, the focal point is noted at the communal attachment to and use of spatial (context-specific environmental) resources to manage (the risk of) flood hazard. In the following paragraphs, the detailed use of spatial resources (and other specific approaches) is given, including their meaning and implications regarding tackling the impacts and risk of flood.

### Use of context-specific environmental resources for flood risk management

All identified TEAs manifest the fact of using context-specific environment resources to come to terms with environmental events. In the rituals and the planting of cultural flood-immunising indigenous plants, it is noted that certain types of plants and animals are referred to (as used in the process of remedying or preventing the flood disasters). This exemplifies the continuous co-evolution and interaction between nature and humans. This co-evolution and interaction constitutes the local ecologies which explain the spatial relations of humans with their particular environment (Arms 2004; Kottak 2009). The more environmental resources are used, the more humans connect with their environment; accordingly, the better they understand the processes that occur therein and devise ways to address them – it is this connectivity that is exemplified in the use of environmental resources in this study, that is, the use of local ecologies to build on experiences and strengthen resilience (to the assessed flood risk) through designing appropriate measures or response. This way, they learn to live with the environmental processes, for instance, leaving with floods (Hyndman & Hyndman 2014).

The use of environmental resources (and the entire related practices) to come to terms with nature is accustomed to a Bantu (of which the Nyamwamba community in the Rwenzori is part). Referring to the Bantu, Tempels (1959) uses the phrase ‘vital force’ to refer to what was observed among the Bantu: a Bantu taps the vital force or energy (i.e. uses environmental resources) to strengthen their well-being against any misfortunes or calamities. In this case, every specific material touched (i.e. the culturally sensitive plants, the animals scarified and the rituals to remedy the adverse impacts and risk of flooding) is thus termed as vital force to give the needed energy to people who seek it. Then, everything becomes spiritual, in which case the participants must connect with the spiritual to perform the traditional practices. Perhaps, that is why the plants should be planted by a designated or (culturally) qualified person, or the cleansing ritual by the chieftain. Details about each of these practices, and their implications for addressing impact and risk of flood events, are discussed in the following paragraphs.

### The cleansing rituals, cultural plants and flood hazards

The rituals and the planting of cultural plants illustrate how the traditional community has strong attachment to the invisible world. From the science point of view, the invisible world is hard to comprehend, especially when linked with traditional (African) religions which involve a place attachment (as it seems the case for the Rwenzori). It is argued that religious place attachment seems incapable of enhancing preparedness (Mishra, Mazumdar & Suar 2010), perhaps because of the deterministic viewpoint associated with it. Accordingly, rituals linked to it would hardly offer reliable measures to address impacts and risk posed by flood events. However, as different environments nurture different traditional practices (Kottak 2009), every ritual linked to religion may hardly be insensitive to FRM. Already, the situation in the Rwenzori seems different with what is expected to a community believing in the invisible.

Belief in the invisible or religious explanation of the hazards (and consequent disasters) would mean belief in fatalism or determinism and most likely doing nothing, as the spirits would have more power than humans (Smith & Petley 2009). From the point of view of the practice of cleansing, which can be interpreted as a symbolic moral gesture, a fatalistic mindset would seem to be the case for the Rwenzori. Conversely, it is observed that the community does something about the spirits: they can manage and/or chase them away, for instance, through the ritual or planting a certain plant to immunise a certain area against the spirit; also, the traditional hydro-meteorological expert (i.e. rainmaker) uses the ancestral spirit to make or prevent rain. Which sort of spirits are these that obey human actions? Although it is difficult to understand this, there seems to be an implicit pattern – when certain actions are done about the spirits, something happens as result. Thus, whereas these rituals are hardly understood from a scientific point of view, it is paramount to study them to ascertain the science that could be behind them regarding FRM. Of relevance will be the understanding of the ‘denotations’ and ‘connotations’ of the rituals to give technical meanings and relations to FRM, for instance: how does the cleansing exercise start, who stands where, which songs are chanted, which animal species are sacrificed, which words are spoken by leader? Also, how does the diviner study the river dynamics? How does the ‘*rainmaker*’ actually use an ancestral spirit to make rain or does he just forecast weather? Which behavioural aspects exist that can motivate local people to abide by the norms regarding hydrological resources and systems? And, how do all practices relate to modern FRM?

Apparently, whether or not the spirits that are referred to in the rituals (or in the immunising cultural plants) exist, is a question of what they actually mean by ‘spirit’ and the practice of appeasing spirits. Framed from the viewpoint of Tempels (1959), the spirits may not actually mean the invisibles (as could be quickly thought of), but perhaps an ecological aspect. A clear link appears with the indigenous plants that are believed to immunise against flood – the belief that flood disasters would subside if indigenous plants, such as bamboo, are planted along rivers is noted.

Indigenous species (among which is bamboo plants) have been cited to have more incentive for the local [bio]diversity in various indigenous communities. The study of the impact of deforestation on flood risk in developing countries by Bradshaw et al. (2007) is a typical example. According to this study, flood frequency negatively correlated with the amount of the remaining natural forests and positively correlated with loss in natural forest area. The authors concluded that flood risk might escalate because of the unabated loss of natural forests. Unlike exotic forests and tree plantations, indigenous tree species exhibit a high conservation value for indigenous ecological diversity, which enhances their abilities to reduce hydraulic conductivity and intercepting runoff and/or rainfall. Because of their fibrous root, rhizome and extensive leaf systems, (1) they hold soil together and reduce hydraulic conductivity, and (2) their canopies intercept rain water and fibrous roots take-up large quantities of intercepted water and releases it through transpiration. Furthermore, they improve hydrological systems such as the wetlands (these in turn improve the storage of the excess water in the river) – altogether, over time, these indigenous ecological processes strengthen the local riparian biotic and abiotic diversity that make up the riverbanks and hydrological systems and thereby reduce the chances of a flood disaster (Abam 1993; Barbedo et al. 2014; Brody et al. 2007; Calder & Aylward 2006). This would mean that FRM approaches that support planting of the traditional ecological plants and related practices would be most suited to address the impacts and risks posed by floods. Nevertheless, with the change from spontaneous to market economy and market-oriented livelihoods, there is likelihood that a shift from the values attached to the riverine ecosystems services may have occurred (De Hontheim 2015). In this sense, the willingness by the indigenous people to plant indigenous plants would be in doubt. Conversely, this means that incentivising indigenous plant species (Maathai 2010) would still enhance planting them along rivers and catchments. Therefore, holistic empirical investigations on these identified indigenous species could bring new knowledge to flood risk science as a discipline (in addition to providing solutions to the communities-at-risk). Emphasis is required on those cited and other indigenous plants associated with flooding in the Rwenzori whose river ecology is not clearly illustrated in current literature.

### The role of religion and conservationism on the traditional ecological approaches in the Rwenzori

The preceding sections illustrated that TEAs in the Rwenzori could enhance approaches for addressing impact and risk posed by flood hazards. Moreover, the practice of planting indigenous plants is found much consonant with contemporary flood risk science (where planting of trees and preservation of wetlands is now being emphasised). However, religion and conservation of the Rwenzori Mountains ecosystems seems to have continuously provided a setback to the traditional practices.

Various scholars frequently attribute the occurrence of extreme environmental events (in Africa) on the disappearance of traditional approaches that used to address them (Iloka 2016; Maathai 2010; Sillitoe & Marzano 2009). A typical example of how religious campaign against TEAs paved the way to degradation of the Kenya mountain ecosystems is given by Maathai (2010); it is illustrated that the local people were convinced (by the new religion): firstly, that God never stayed in the mountain; secondly, that it was evil to revere the mountain and its resources; and thirdly, there was no harm if they violated the traditional precepts regarding the mountains ecosystem. Accordingly, the motive and sense of place attachment, which enabled indigenous communities to care for the mountain ecosystems to enhance their livelihoods was disenfranchised.

What has the practice of a Christian region to do with TEAs? This study shows that TEAs are not about the practice of a religion in the perspective of the modern religion. It is noted that the ‘*spirits*’ talked about are not religious; they are, rather, ecological phenomena (that traditional communities fail to understand). Most importantly, TEAs are geared to maintaining a livelihood through conserving the environment which provides the ecosystems services. To ensure continuous ecosystem provisioning services, the community people conducted the relevant practices (TEAs). As such, TEAs are a matter of respect and fairness to environment which was regarded as provider of a living. Perhaps, it is this approach which was wrong in the religious sense as it would be related to idolatry (Muhumuza 2015) or in the sense that God, not nature in the strictest sense of the term, is regarded as the provider to humanity. Then, maybe, it should have been interpreted that (1) God never stays in the mountains, but provides to the people through the mountain ecosystems, and (2) that it was respect to God to conserve that creation that God made (i.e. the ecosystem resources). This interpretation is also implied in one of the recent encyclicals, indicating the duty of the (local) people to conserve creation (Bergoglio 2015). Considering that such encyclicals are recent, it is probable that the religious teaching on TEAs will be considerate; and so is the reason studies on the use of such practices in FRM should involve participants from the religious side(s).

Related to the religious issue is the view that *successful conservation should be devoid of human interactions*: the human activities in conservation areas parks are frequently seen as detrimental to successful conservation (Muhumuza & Balkwill 2013). Accordingly, human acts in the conservation areas that would be geared towards FRM are denied. However, it remains sceptical if a community that energises life through the environmental resources would not wish to conserve those very resources. One argument against this would be on population increase.

Human population increase is echoed as a seed for environmental degradation. Quite famous regarding this aspect is the concept of the ‘*tragedy of the commons*’ as advanced (Hardin 1968). In this concept, as humans increase, their freedom to use common environmental resources turns to degradation; hence, more human powers must be given up to ensure environmental conservation. Maathai (2010) re-echoes this and argues that humans sometimes have a low incentive to think beyond immediate needs when it comes to utilisation of environmental resources; thus, the needs to conserve are influenced by whether the immediate needs are met. Then, with increase in population as reported in these Rwenzori communities (UBOS 2016; UNDP 2013), exhaustion would be predicted; frequently practices would perhaps take place to harness provisioning services to sustaining livelihoods. However, it seems sufficient to infer that the increased need for provisioning services would serve as an incentive to conserve the resources; except if a drive would be made to change the nature of the natural system to suit the increasing demand for the services. Besides, considering that the TEAs to flooding in the Rwenzori have not been given a clear scientific investigation, it is relevant to understudy what would be their influence FRM (in particular).

Even though further investigations are required, it can be inferred that TEK (and the linked TEAs) are *primarily* neither detrimental to conservation nor opposed to Christian region. They are just ways of maintaining a livelihood through preserving the provisioning services of the environment: indigenous community way of life used to reduce disaster and enhance a good life. Thus, both the religious and conservationist view of the TEAs might be either a question of difference in epistemic orientations or misunderstanding the traditional way of life of these very indigenous people. Therefore, incorporating relevant aspects of the TEAs into FRM requires an epistemic illustration that there is no compromise of either religion or conservation despite the short-comings that might come with the TEAs themselves. Thus, studies on the TEAs and their incorporation into FRM need to involve the two institutions.

## Conclusion

In the Rwenzori, the existence of the TEK is framed in various TEAs. An interpretation is proposed to link TEAs to effective FRM in the Nyamwamba watershed (Rwenzori Mountains) where on 01 May 2013 floods caused deadly impacts. More specifically, this study has (1) documented the TEAs to flooding that were used in the past, (2) highlighted those that are still relevant to contemporary flood risk science and (3) illustrated their relevance to effectively manage flooding in the Rwenzori. From the TEAs, the most relevant to contemporary flood science include (1) the planting of culturally immunising indigenous species and (2) the ritual of mountain cleansing practice which appears to have been used as an awareness-raising platform. Indigenous species are important for improving hydrological and riparian systems, thereby reduce the chances of flooding (compared to species that are alien to riverine catchments). Although the ritual practices were found difficult to understand from a scientific epistemic point of view, it is noted that there might exist some undiscovered science(s) because of the seeming pattern behind them; for instance, the diviner and rainmaker seem related to hydrological prediction and weather forecasting respectively. Considering that this study only confirms the existence of TEAs to flooding in Rwenzori, a full-scale study of each TEA will be relevant to effective FRM in the Rwenzori in particular and to flood risk science in general. In the full-scale study of the TEAs, it will be interesting to involve the religious and conservationists. More specifically, an epistemic illustration is relevant to indicate how the TEAs and their associated spirits are not religious, but rather ecological and that they compromise neither religious doctrine nor conservation (at least in the primary sense).

## Footnotes

1 Indigenous knowledge is that body of *native worldviews* (cosmovision) used by citizens of indigenous communities to survive life stresses through generations (Iloka, 2016; Muhumuza, 2015).
